# Supplementary material for: Sirt6 Activation Ameliorates Inflammatory Bone Loss in Ligature-Induced Periodontitis in Mice
Source: Int J Mol Sci. 2023 Jun 27;24(13):10714. doi: 10.3390/ijms241310714 (PMC10341680; doi:10.3390/ijms241310714)
Supplement: Supplementary file 1 [file ijms-24-10714-s001.zip › ijms-2450964-supplementary.pdf]

Sirt6 Activation Ameliorates Inflammatory Bone Loss in Ligature-Induced Periodontitis in Mice

Myung Jin Lee [1,†], Hyang Hwa Ryu [2,†], Jae Won Hwang [2], Jung Ryul Kim [3], Eui-Sic Cho [4], Jin Kyeong Choi [5,*] and Young Jae Moon [2,3,*]

1 Department of Chemistry, Case Western Reserve University, Cleveland, OH 44106, USA; jinalee939@gmail.com
2 Department of Biochemistry and Molecular Biology, Jeonbuk National University Medical School, Jeonju 54896, Republic of Korea; yy-ryu@hanmail.net (H.H.R.); toriake11@naver.com (J.W.H.)
3 Department of Orthopaedic Surgery, Jeonbuk National University Medical School and Hospital, Jeonju 54896, Republic of Korea; jrkeen@jbnu.ac.kr
4 Cluster for Craniofacial Development and Regeneration Research, Institute of Oral Biosciences, Jeonbuk National University School of Dentistry, Jeonju 54896, Republic of Korea; oasis@jbnu.ac.kr
5 Department of Immunology, Jeonbuk National University Medical School, Jeonju 54907, Republic of Korea
* Correspondence: jkchoi@jbnu.ac.kr (J.K.C.); yjmoonos@jbnu.ac.kr (Y.J.M.)
† These authors contributed equally to this work.

Keywords: alveolar bone loss; inflammation; MDL801; mouse models; osteoclast; periodontitis; Sirt6

1. Supplementary Figure

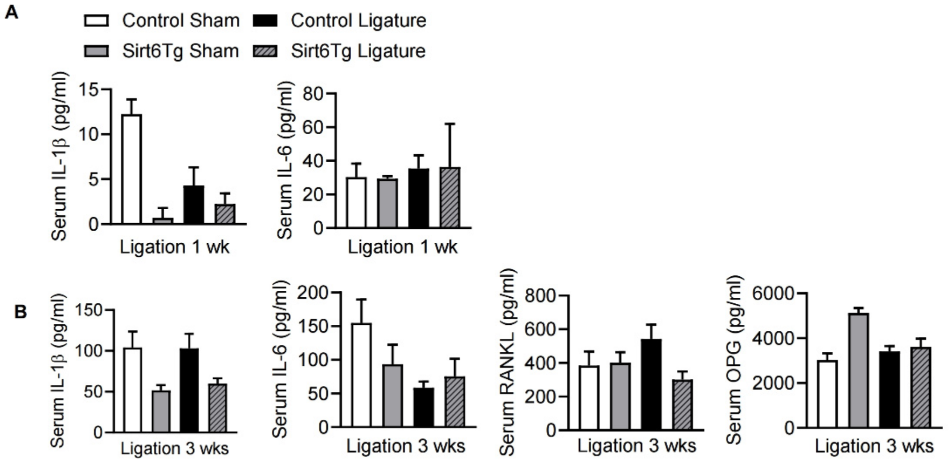

Supplement Figure S1. Systemic inflammatory cytokines did not change in the ligature-induced periodontitis model. ELISA analysis of IL-1β, IL-6, RANKL, and OPG in serum of control sham, control ligature, Sirt6Tg sham, and Sirt6Tg ligature (n=3–5).
